# The 19th EML as a global standard: an innovative approach or just an opportunity for new and effective medicines?

**DOI:** 10.1186/2052-3211-8-S1-O14

**Published:** 2015-10-05

**Authors:** Nicola Magrini, Lorenzo Moja, Bernadette Cappello, Kees De Joncheere, Gilles Forte

**Affiliations:** 1 Essential Medicines and Health Products Department (EMP), World Health Organization (WHO), 1211 Geneva 27, Switzerland

## 

In 1977, the World Health Organization (WHO) published its first Model List of Essential Medicines including just over 200 medicines. This year, the 20th Expert Committee for the Selection and Use of Medicines has included several new high priced medicines in the areas of cancer (N=16), hepatitis C (N=6) and multidrug-resistant tuberculosis (N=4).

The large cancer update was prepared with the support of UICC by an ad-hoc expert working group that reviewed the overall consistency of 29 applications to WHO EML. This large update challenges the traditional perception of essential medicines emphasizing the role of EML as a global standard and as an important tool to prioritise medicines reimbursement, as part of UHC programs in countries.

The Expert Committee's main criteria for inclusion in the List were public health relevance, the magnitude of clinical benefit and a favourable risk–benefit profile determined through a systematic method of evidence synthesis and appraisal. Estimates of the magnitude of the benefit are particularly pertinent for medicines for cancer, given the small gains in life expectancy offered by some new and expensive treatments that led to the decision to select only a limited range of new cancer medicines. The Expert Committee could not suggest a minimum threshold of benefit for cancer medicines because this is out of its role and mandate, but suggested the need for a medium-term strategy to re-discuss existing mechanisms in particular to present approaches and thresholds for both clinical relevance and cost-effectiveness.

The inclusion in EML of several highly effective and high priced medicines is raising important questions of how access and affordability can be addressed at global and country levels. The existing EML decision-making rules in place since the EB resolution of 2001 state that absolute treatment cost should not be a reason to reject a proposed addition to the Model List if criteria for benefit and public health relevance are met. In practice, affordability has been changed from a precondition for listing an essential medicine to a challenge that must be addressed once an effective and expensive medicine has been added to the list.

The 20^th^ Expert Committee advocated for new and better-coordinated actions at a global level to address issues of thresholds for clinical benefit and cost-effectiveness. Prioritization according to the magnitude of benefit was the guiding principle for EML decision-making and could be relevant to countries in their reimbursement decisions and in developing universal health coverage programs.

